# Effect of ketamine on reward processing in depressive disorders: a systematic review of neuroimaging studies

**DOI:** 10.1017/S109285292610087X

**Published:** 2026-03-10

**Authors:** Halima Faisal, Gia Han Le, Angela T.H. Kwan, Sabrina Wong, Will Cheung, Christine E. Dri, Bing Cao, Taeho Greg Rhee, Stavroula Bargiota, Heidi K.Y. Lo, Bianca Shen, Hernan F. Guillen-Burgos, Roger S. McIntyre

**Affiliations:** 1 Bachelor of Medical Sciences, Department of Schulich Medicine and Dentistry, Western University, London, ON, Canada; 2 Centre for Addiction and Mental Health, Toronto, ON, Canada; 3 Brain and Cognition Discovery Foundation, Toronto, ON, Canada; 4 Institute of Medical Science, Temerty Faculty of Medicine, University of Toronto, Toronto, ON, Canada; 5 Poul Hansen Family Centre for Depression, University Health Network, Toronto, ON, Canada; 6 Faculty of Medicine, University of Ottawa, Ottawa, ON, Canada; 7 Department of Pharmacology & Toxicology, University of Toronto, Toronto, ON, Canada; 8 Faculty of Psychology, Southwest University, Chongqing, China; 9 Department of Psychiatry, Yale University School of Medicine, New Haven, CT, USA; 10 Department of Psychiatry, University of Thessaly, Larissa, Greece; 11 Department of Psychiatry, School of Clinical Medicine, LKS Faculty of Medicine, The University of Hong Kong; 12 Department of Health Sciences, Queen’s University, Kingston, ON, Canada; 13 Pontificia Universidad Javeriana, Department of Psychiatry and Mental Health, Bogotá, DC, Colombia; 14 Universidad Simon Bolivar, Center for Clinical and Translational Research, Barranquilla, Colombia; 15 Center for Clinical and Translational Research, Bogotá, DC, Colombia; 16 Department of Psychiatry, University of Toronto, Toronto, ON, Canada

**Keywords:** Anhedonia, depression, glutamate, ketamine, plasticity, reward, therapeutics

## Abstract

**Background:**

Anhedonia and reward-processing deficits are core features of major depressive disorder (MDD) that respond poorly to traditional antidepressants. Ketamine has rapid antidepressant effects, yet its neurofunctional actions within reward circuits remain unclear. We synthesized human neuroimaging evidence on ketamine-related modulation of reward circuitry and implications for anhedonia.

**Methods:**

Following Preferred Reporting Items for Systematic Reviews and Meta-Analysis (PRISMA) guidelines, we searched Ovid Embase/MEDLINE/PsycINFO, Cochrane Library, Scopus, Web of Science, and Google Scholar. Eligible studies included adults with MDD receiving ketamine or esketamine and undergoing fMRI, PET, or related imaging during rest or reward/emotion tasks. Thirteen studies met inclusion criteria (N = 623; 482 MDD/TRD, 141 controls), mostly randomized, double-blind, and placebo-controlled; no eligible esketamine neuroimaging studies were identified.

**Results:**

Intravenous ketamine (typically 0.5 mg/kg over 40 min) was associated with short-term modulation of fronto-striatal and limbic networks. Resting-state fMRI commonly showed altered ventral striatal–prefrontal/ACC connectivity and broader DMN/salience/executive network reorganization across acute-to-subacute windows (≈2–48 h), with some effects changing at later follow-up (≈10 days). Task-based fMRI showed altered ventral striatal/putaminal responses during reward anticipation/feedback and modulation of medial prefrontal/cingulate activity during emotion processing. PET findings suggested increased prefrontal–cingulate metabolism and region-specific 5-HT₁B binding/availability changes, with baseline ventral striatal 5-HT₁B measures associated with symptom profiles and symptom change.

**Conclusions:**

Ketamine is associated with rapid reconfiguration of reward-related circuitry, but few studies directly measured anhedonia; findings likely reflect broader reward-processing and antidepressant-associated mechanisms. Larger longitudinal multimodal studies are needed to validate biomarkers and durability.

## Summations

- **Resting fMRI:** Ketamine was associated with fronto-striatal/ACC connectivity changes (direction varied by circuit and dose).
- **Task fMRI:** Ketamine was associated with higher striatal reward feedback responses and mPFC/ACC modulation.
- **PET:** PET suggested dose-related ACC/PFC metabolic/connectivity effects and region-specific 5-HT1B binding changes.

## Limitations

- Heterogeneity across modalities and tasks, as each captures different aspects of neural function (evoked versus intrinsic/spontaneous activity), limiting direct comparability.
- Methodological variability and participant heterogeneity may have influenced results.
- Most imaging assessments were acute/subacute with limited follow-up, restricting inference about durability of neural changes and relationships to longer-term outcomes.
- Only a minority of studies used validated anhedonia-specific scales, and many studies had modest sample sizes, limiting symptom-specific interpretation and generalizability.

## Introduction

Major depressive disorder (MDD) is one of the leading contributors to global disability and disease burden, with an estimated lifetime prevalence of ~15% worldwide.^1^^,^ ^2^ MDD is characterized by persistent low mood, diminished energy, and anhedonia—marked reduction in the ability to experience pleasure or interest in previously enjoyable activities—which negatively impacts quality of life, interpersonal relationships, and overall homeostatic functioning.^3^^–^^9^

Replicated neuroimaging evidence suggests that disturbances within reward-related brain regions, particularly the prefrontal cortex and mesocorticolimbic structures, are implicated in individuals with MDD.^6^^,^^10^^,^^11^ The mesocorticolimbic dopamine system, comprising the ventral tegmental area (VTA), nucleus accumbens (NAcc), ventral striatum, and prefrontal cortex (PFC), has a central role in mediating reward-related processes.^12^^–^^15^ Task-based functional magnetic resonance imaging (fMRI) studies have consistently demonstrated reduced activation in the ventral striatum and NAcc during reward paradigms in MDD,^16^^,^^17^ alongside altered fronto-striatal connectivity during reward processing tasks.^18^^–^^20^

Preclinical studies have revealed activity of the NAcc is dependent on the reward-association learning process, confirming that dopaminergic D1 receptor modulation in medial NAc supports motivation for both natural and drug rewards.^21^^,^^22^ Notwithstanding advances in pharmacotherapy, conventional monoaminergic antidepressants (eg selective serotonin reuptake inhibitors and serotonin-norepinephrine reuptake inhibitors) have limited efficacy in alleviating and/or ameliorating reward-processing, hedonic capacity and motivational drive impairments.^23^^–^^26^ The aforementioned limitations are especially pronounced in individuals with treatment-resistant depression (TRD) (ie a subgroup of patients exhibiting an inadequate response to at least two antidepressant trials), underscoring the need for mechanistically informed pharmacotherapeutics that target reward-related pathophysiology.^27^^–^^30^ Ketamine, an N-methyl-D-aspartate (NMDA) receptor antagonist, has rapid antidepressant effects with unique mechanistic properties in persons with depression.^31^ Importantly, clinical evidence indicates that its efficacy is not inferior to electroconvulsive therapy in treatment-resistant depression, and accumulating data further demonstrate that ketamine rapidly improves outcomes in TRD and confers significant benefits on patient-reported outcomes such as quality of life and functioning.^32^

While the precise mechanisms underlying ketamine’s rapid therapeutic effects remain incompletely elucidated, prevailing hypotheses emphasize modulation of glutamatergic transmission and enhancement of synaptogenesis.^9^^,^^33^^–^^37^ Early studies using a subanesthetic dose of ketamine (and esketamine) found a rapid and transient increase in glutamate release in the mPFC, believed to initiate activity-dependent release of brain-derived neurotrophic factor (BDNF) and stimulation of protein synthesis–dependent synapse formation, essential for ketamine’s antidepressant effects.^38^^–^^40^ Preliminary evidence suggests that ketamine may normalize frontostriatal and limbic network overactivation in TRD, thereby restoring reward responsiveness and alleviating core symptoms such as anhedonia.^41^^–^^43^ Notwithstanding emerging evidence for ketamine’s pro-hedonic effects,^9^^,^^34^ the neurofunctional mechanisms that mediate these therapeutic effects remain incompletely understood. Herein, the present systematic review aims to (1) investigate ketamine’s neurofunctional effects on reward processing circuits across neuroimaging modalities and (2) evaluate how ketamine’s modulation of reward-related brain circuits may inform mechanistic models of anhedonia in depression.

## Methods

### Search strategy and reporting criteria

This systematic review was conducted following the PRISMA guidelines.^28^^,^^44^ A comprehensive systematic search was performed across Ovid databases (ie Embase, MEDLINE, PsycINFO), Wiley Cochrane Library, Scopus, Web of Science, and Google Scholar. The search strategy utilized a combination of Medical Subject Headings terms and free-text keywords to capture all relevant studies. Search terms were structured around four key concepts, including depression and anhedonia, ketamine, reward processing, and neuroimaging modalities. No language restrictions were applied. Database searches were conducted from February 2025 (inception) to September 2025, with an update on September 2, 2025. The protocol was not registered; however, eligibility criteria and outcomes were defined a priori and were not modified during screening, extraction, or synthesis.

### Inclusion and exclusion criteria

Studies were included if they met the following criteria: (1) human participants aged 18–65 years diagnosed with MDD (including treatment-resistant depression where specified) based on standard clinical assessment or structured diagnostic interviews, (2) single/repeat-dose intravenous, oral, or intranasal ketamine or esketamine administration as the primary intervention, (3) prospective interventional studies, including randomized controlled trials (parallel or crossover), non-randomized controlled trials, and open-label/single-arm interventional studies that used validated clinical scales to assess depression severity, (4) used neuroimaging using fMRI (resting-state and/or task-based), positron emission tomography (PET), fluorodeoxyglucose positron emission tomography (FDG-PET), pharmacological magnetic resonance imaging (phMRI), or related modalities, assessing reward- and/or positive-valence–relevant circuitry during reward/emotion tasks or at rest, and (5) clearly reported timing of imaging in relation to ketamine/esketamine administration (eg during infusion and/or post-dose timepoints). Studies were excluded if they were case reports/series, reviews/meta-analyses, non-human studies, or purely observational designs without ketamine/esketamine administration.

### Data extraction

Data extraction was conducted independently by two reviewers (H.F. and W.C.), and discrepancies were resolved through consensus. The following information was extracted from included articles: first author, sample size and demographics, study design, ketamine intervention details (dose, route, and timing), type of neuroimaging modality, reward-related regions of interest, and measures of reward outcomes (Table 1).

**Table 1.** Summary of Included Studies Evaluating Ketamine’s Effects on Reward Processing Across Neuroimaging Modalities

First Author, Year	Sample size (n)	Age (SD)	% Female	Dosage & route	Comparator	Modality & scale(s)	Timing of scan	Reward outcomes
**Resting-state fMRI studies**								
Evans et al., 2018^45^	MDD: 33, HC: 25	MDD: 36 (10), HC: 33 (10)	MDD: 61, HC: 60	0.5 mg/kg, IV over 40 minutes	Placebo; 0.9% saline	fMRI, MADRS	Five fMRI scans per subject: baseline (2–3 days before first infusion), post-placebo (day 2–3 and day 10–11), and post-ketamine (day 2–3 and day 10–11).	Ketamine transiently increased insula–DMN connectivity at 48 hours compared with placebo (*p* < .05), which returned to baseline by day 10.
McMillan et al., 2019^46^	MDD: 26	30.2 (8.2)	50	0.25 mg/kg bolus +0.25 mg/kg, IV over 45 minutes	Placebo; Remifentanil	fMRI, MADRS	Ketamine was administered 7 minutes into the 16-minute rsfMRI	Ketamine significantly increased BOLD signal in ACC, paracingulate gyrus, superior frontal gyrus, and right insula, while decreasing signal in sgACC, mPFC, hippocampus, amygdala, and putamen relative to placebo (*ps* < .05).
Chen et al., 2019^47^	0.5 mg/kg: 16, 0.2 mg/kg: 16, placebo: 16	0.5 mg/kg: 43.3 (11.9), 0.2 mg/kg: 44.4 (10.8), placebo: 49.9 (8.1)	0.5 mg/kg: 69 0.2 mg/kg: 69 placebo: 81	0.2 mg/kg and 0.5 mg/kg IV over 40 minutes	Placebo; 0.9% saline	fMRI, MADRS	baseline before ketamine injection and the third day after ketamine injection (48 h)	At 0.5 mg/kg, ketamine reduced FC between left dACC–right ACC and right dlPFC–frontal pole, while at 0.2 mg/kg it decreased FC between dACC and multiple frontal/parietal regions and increased FC between dlPFC–superior parietal lobe and dACC–middle temporal gyrus (all *ps* < .05).
Mkrtchian et al., 2021^41^	TRD: 33, HC: 25	TRD: 36 (9.54), HC: 34 (10.97)	TRD: 60, HC: 67	0.5 mg/kg, IV (time NR)	Placebo: 0.9% saline	fMRI, SHAPS & MADRS	fMRI scans were obtained 2 days after each infusion (crossover sessions 2 weeks apart)	Ketamine increased connectivity between ventral striatal seeds and dlPFC, vlPFC, pgACC, and OFC in patients with depression but decreased connectivity in healthy controls (*ps* < .05).
Alexander et al., 2023^48^	TRD: 29, HC: 21	TRD: 36 (10), HC: 35 (11)	TRD: 62, HC: 62	0.5 mg/kg, IV over 40 minutes	Placebo: 0.9% saline	fMRI, MADRS & SHAPS & TEPS	fMRI scans were obtained 2 days after each infusion (2 weeks apart)	In the TRD group, ketamine significantly increased sgACC connectivity with bilateral pgACC, anterior vmPFC, and right ventral striatum, while decreasing connectivity with the right hippocampus/parahippocampal gyrus (*p* < .05).
**Task-based fMRI Studies**								
Murrough et al., 2015^49^	TRD: 18, HC: 20	TRD: 38.1 (13.8), HC: 35 (8.9)	TRD: 44.4, HC: 45	0.5 mg/kg, IV over 40 minutes	Healthy controls	fMRI, MADRS	Baseline (Time 1) and 24 hours following a single intravenous infusion of ketamine (Time 2).	Ketamine increased right caudate responses to happy vs neutral faces (Emotion × Time interaction, cluster-level FWE *p* < .05).
Reed et al., 2018^50^	TRD: 33, HC: 26	TRD: 36.1 (9.7) HC: 33.9 (10.4)	TRD: 64 HC: 62	0.5 mg/kg, IV over 40 minutes	Placebo; 0.9% saline	fMRI, MADRS	Baseline scan, 95% of scans took place 2 days post-infusion (1–3 days), then 85% of scans took place 11 days after infusion (9–13 days)	Ketamine increased activation in the left middle occipital gyrus (*p*FWE < .001) but reduced activation in the left temporal and inferior frontal cortices (*p*FWE < .002) compared with placebo.
Reed et al., 2019^51^	TRD: 33, HC: 24	TRD: 35.9 (9.8), HC: 34.4 (10.7)	TRD: 61, HC: 63	0.5 mg/kg, IV over 40 minutes	Placebo: 0.9% saline	fMRI, MADRS	Baseline scan, then fMRI 1–3 post infusion (95% at day 2); infusions were 2 weeks apart	In MDD, ketamine led to widespread reductions in bilateral frontal, temporal, precuneus, and posterior cingulate activity compared with placebo (*p*FWE < .001), while in healthy controls it reduced bilateral cingulate activity but increased a large frontal cluster (*p*FWE < .001).
Kotoula et al., 2022^52^	rMDD: 37	Total: 28.5 (NR)	57	0.5 mg/kg, IV over 40 minutes	Placebo: 0.9% saline	fMRI, SHAPS	Ketamine and saline were administered during a 40-minute steady-state infusion, and the sessions were at least 7 days apart. Participants were scanned 2 hours after the end of the infusion.	Ketamine increased activation across reward ROIs during anticipation of win vs neutral trials (*F*1,36 = 9.261, *p* = .003), and significantly increased NAc and putamen activity during feedback of low-win trials vs neutral (*p*CORR = .008).
**Other Neuroimaging Studies**								
Downey et al., 2016^53^	Ketamine: 21, placebo: 19	Ketamine: 27.1, placebo: 25.7	ketamine: 61.2, placebo: 57.9	0.5 mg/kg, IV over 60 minutes	Placebo: 0.9% saline	phMRI, MADRS & BDI	phMRI scans were collected 5 minutes before and for 40 minutes during infusion	Ketamine significantly increased sgACC and rACC BOLD responses compared with placebo during infusion (*p*FWE < .05), with additional activation in caudate and thalamus.
Chen et al., 2017^54^	0.5 mg/kg: 8, 0.2 mg/kg: 8, placebo: 8	0.5 mg/kg: 51.13 (13.59), 0.2 mg/kg: 49.75 (11.8), placebo: 46.25 (8.14)	0.5 mg/kg: 100, 0.2 mg/kg: 62.5, placebo: 62.5	0.2 mg/kg and 0.5 mg/kg, IV over 40 minutes	Placebo: 0.9% saline	FDG-PET, HDRS–17	The first scan was performed immediately before a single dose of ketamine infusion. The second scan was performed 1 day after the ketamine infusion.	Patients receiving 0.5 mg/kg ketamine showed increased SUV in SMA and dACC compared with 0.2 mg/kg ketamine (*p* = .014). dACC SUV increase was negatively correlated with depressive symptoms at day 1 (P = 0.034),
Tiger et al., 2020^55^	Ketamine: 20, Placebo: 10	Ketamine: 39.2 (NR), Placebo: 37.1 (NR)	Ketamine: 40, Placebo: 60	0.5 mg/kg, IV over 40 minutes	Placebo: 0.9% saline	T1 MRI + PET & MADRS	PET1 at baseline; treatment administered 1–3 days after PET1. PET2 acquired 24–72 hours post-treatment (typically the day after infusion).	No significant group×time difference in BPND between ketamine vs placebo across ROIs. Hippocampus BPND increased ~16.7% after ketamine. Baseline VST BPND correlated inversely with baseline depression severity and inversely with symptom improvement after ketamine.
Kopelman et al., 2023^56^	Ketamine: 67, Placebo: 31	Range: 18–60	Ketamine: 59.70, Placebo: 67.74	0.5 mg/kg, IV over 40 minutes	Placebo: 0.9% saline	DTI, MADRS & QIDS-SR	Pre-infusion baseline and 24 hours post-infusion	Gray-matter mean diffusivity changes from baseline to 24 hours post-infusion were associated with depression improvement. Effects were largely driven by ketamine in some ROIs (left BA10 and left amygdala)

MDD = major depressive disorder, HC = healthy controls, TRD = treatment-resistant depression, rMDD = remitted major depressive disorder, IV = intravenous, fMRI = functional magnetic resonance imaging, rsfMRI = resting-state functional magnetic resonance imaging, phMRI = pharmacological magnetic resonance imaging, FDG-PET = fluorodeoxyglucose positron emission tomography, PET = positron emission tomography, T1 MRI = T1-weighted magnetic resonance imaging, DTI = diffusion tensor imaging, MADRS = Montgomery–Åsberg Depression Rating Scale, BDI = Beck Depression Inventory, HDRS-17 = 17-item Hamilton Depression Rating Scale, QIDS-SR = Quick Inventory of Depressive Symptomatology–Self-Report, SHAPS = Snaith-Hamilton Pleasure Scale, TEPS = Temporal Experience of Pleasure Scale, BOLD = blood-oxygen-level-dependent, FC = functional connectivity, DMN = default mode network, ACC = anterior cingulate cortex, dACC = dorsal anterior cingulate cortex, sgACC = subgenual anterior cingulate cortex, pgACC = pregenual anterior cingulate cortex, rACC = rostral anterior cingulate cortex, mPFC = medial prefrontal cortex, vmPFC = ventromedial prefrontal cortex, dlPFC = dorsolateral prefrontal cortex, vlPFC = ventrolateral prefrontal cortex, OFC = orbitofrontal cortex, NAc = nucleus accumbens, ROI/ROIs = region(s) of interest, FWE = family-wise error, pFWE = family-wise error–corrected p value, pCORR = corrected p value, SUV = standardized uptake value, SMA = supplementary motor area, BPND = binding potential (non-displaceable), VST = ventral striatum, BA10 = Brodmann area 10, NR = not reported, mg/kg = milligrams per kilogram.

## Results

### Search results

**Figure 1.**
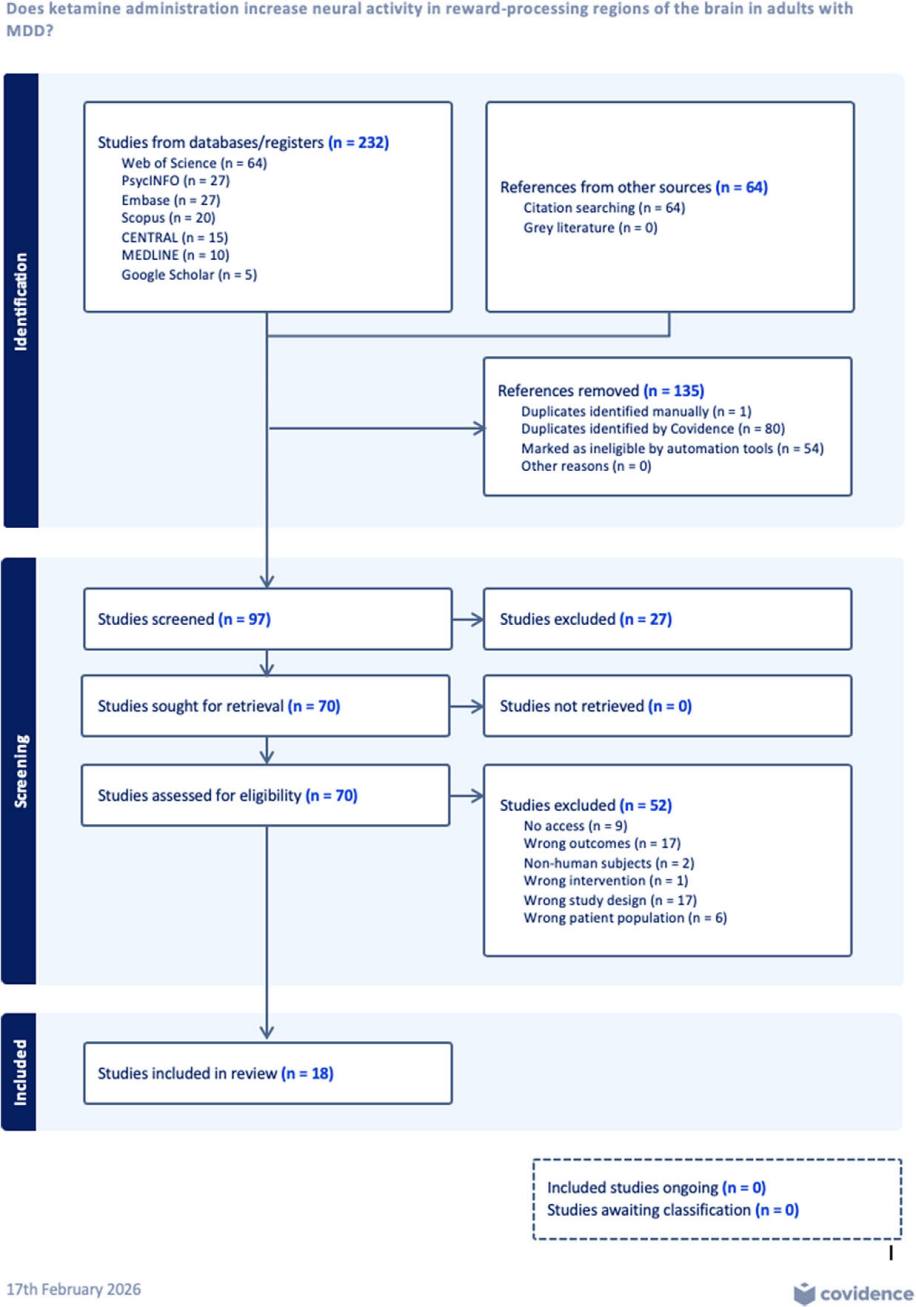
PRISMA flow diagram showing the study selection process.

The initial search identified a total of 232 articles (Figure 1). Additionally, 64 studies were identified through citation searching. After the removal of 135 duplicate and ineligible records, title and abstract screening were conducted for 97 articles (n = 27 articles excluded). A total of 70 studies were sought for full-text review, and 52 studies were excluded for the following reasons: inaccessibility (n = 9), wrong outcomes assessing other brain circuits or using irrelevant clinical measures (n = 17), non-human subjects (n = 2), wrong intervention (n = 1), wrong study design (n = 17), and wrong patient population (n = 6). Subsequently, 18 studies met eligibility criteria and were assessed for risk of bias; 13 studies were included in data extraction and qualitative synthesis (Table 1).

### Risk of bias assessment

**Figure 2.**
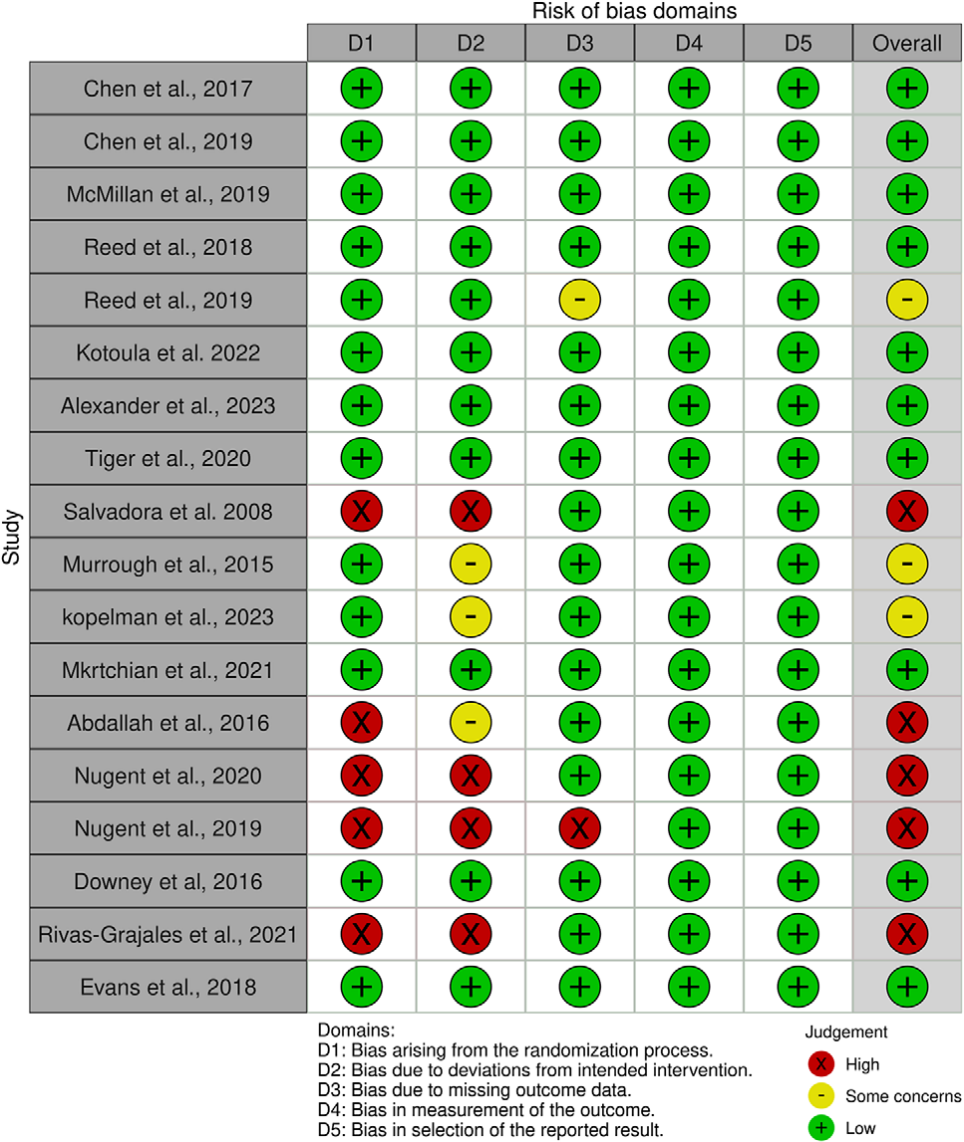
Risk of bias assessment of included studies using Cochrane’s RoB 2.0 tool and visualized using the RobVis R package (McGuinness and Higgins, 2021)^61^.

Most of the studies included in this systematic review were randomized, double-blind, placebo-controlled crossover trials, and the majority of them were evaluated as having an overall low risk of bias. Risk of bias was assessed using the Cochrane Risk of Bias 2.0 (RoB 2.0) tool by two reviewers (H.F. and W.C.) for all studies that met eligibility criteria (n = 18). Most studies were determined to have low risk of bias across domains, particularly concerning missing outcome data, measurement of outcomes, and selection of reported results (domains 3–5). However, five studies^16^^,^^57^^–^^60^ were judged to have a high risk of bias, primarily due to concerns related to the randomization process and/or blinding and were therefore excluded from data extraction and synthesis in accordance with our approach to prioritize internal validity. Figure 2 summarizes RoB assessments for all studies assessed (n = 18), including those excluded from synthesis due to high risk of bias.

### Study characteristics

Thirteen human neuroimaging studies were included in data extraction and synthesis. Most used randomized, double-blind designs (predominantly placebo-controlled) (n = 10), including several double-blind crossover trials with ketamine versus 0.9% saline, active placebo (remifentanil) (n = 1), and one trial comparing ketamine with lanicemine and saline. Across studies, ketamine was administered almost exclusively intravenously, most commonly 0.5 mg/kg over ~40 minutes, with a small number of dose/infusion variations (eg 0.2 mg/kg arms in two studies; bolus+infusion protocols totaling 0.5 mg/kg; and a 60-minute infusion in one study). Across studies, the most common imaging modality was fMRI (n = 9). Studies also utilized phMRI (n = 1), FDG-PET (n = 2), T1-weighted MRI (n = 1), and diffusion tension imaging (n = 1) to assess changes in reward-processing regions post-ketamine. Task-based paradigms were used in four studies, while resting-state or non-task approaches were used in the remaining studies. The total sample size across the included studies was 623 participants (62% female), with 482 participants diagnosed with a depressive disorder diagnosis (MDD/TRD /remittent MDD) undergoing ketamine administration and 141 healthy controls (Table 1). Various clinical diagnostic tools were used to assess depressive symptoms (Montgomery–Åsberg Depression Rating Scale [MADRS], Beck Depression Inventory, Quick Inventory of Depressive Symptomatology – Self-Report, and Hamilton Depression Rating Scale (17-item version)) as well as anhedonia symptoms (Snaith-Hamilton Pleasure Scale [SHAPS] and Temporal Experience of Pleasure Scale [TEPS]). Only a minority of studies (n = 3) employed validated anhedonia-specific measures, whereas the majority relied on global depression scales. Therefore, not all reported neural changes can be directly attributed to changes in anhedonia, and interpretations in the review distinguish between these measurement approaches. Neuroimaging timepoints clustered into three main windows: during infusion (phMRI/rsfMRI), acute post-infusion assessments ranging from ~2 hours to ~48 hours, and a smaller subset with extended follow-up out to approximately 10–11 days post-infusion. Although esketamine was eligible, no neuroimaging studies administrating esketamine met inclusion criteria. Characteristics and findings of included studies assessing ketamine’s effects on neural reward processing in adults with MDD, with some studies involving a healthy control group.

### Findings from resting-state fMRI

Most rsfMRI studies used 0.5 mg/kg IV ketamine infused over 40 minutes in crossover designs, with scanning conducted at baseline and approximately 2 days post-infusion.^41^^,^^45^^,^^48^ One additional longitudinal resting-state study in TRD compared 0.5 mg/kg vs 0.2 mg/kg vs placebo, with scans acquired at baseline and day 3 post-infusion.^47^ In TRD, ketamine was associated with increased sgACC connectivity to bilateral pgACC, bilateral anterior vmPFC, and right ventral striatum relative to placebo, while decreasing sgACC connectivity to the right hippocampus/parahippocampal gyrus (*p* < .05).^48^ Within the TRD subgroup, changes in sgACC–pgACC connectivity following ketamine versus placebo were associated with improvement in SHAPS scores, suggesting a symptom-relevant link between sgACC coupling and hedonic function. Similarly, ketamine increased functional connectivity between ventral striatal seeds and the dorsolateral prefrontal cortex (dlPFC), ventrolateral PFC (vlPFC), pregenual ACC, and orbitofrontal cortex (OFC) in patients with depression, whereas connectivity decreased in healthy volunteers.^41^ Within TRD, improvement in SHAPS scores at day 2 was associated with increased dorsal caudate–right vlPFC connectivity, while day 10 SHAPS improvement was associated with increased dorsal caudate–pgACC connectivity. Findings across multiple studies also indicated altered connectivity within the default mode network (DMN), salience network (SAL), and central executive network (CEN), which encompass reward-related prefrontal and cingulate regions.^45^^,^^56^ In TRD patients randomized to different ketamine doses, the standard-dose group (0.5 mg/kg) demonstrated reduced functional connectivity of the left dACC and right dlPFC with other frontal regions, while the low-dose group (0.2 mg/kg) showed a broader reduction in bilateral dACC connectivity with frontal and parietal regions.^47^ Within the standard-dose group, reductions in suicidal ideation were negatively correlated with reductions in connectivity between the left dACC and right ACC, indicating symptom-linked connectivity associations. Within the low-dose group, reductions in suicidal ideation were positively correlated with increased connectivity between the right dlPFC and left superior parietal region, suggesting dose-sensitive connectivity–symptom relationships. In a simultaneous EEG/fMRI infusion study, ketamine was associated with increased BOLD signal in ACC, paracingulate gyrus, left frontal pole, superior frontal gyrus, parahippocampal gyrus, and right insula.^46^ Ketamine was also associated with decreased BOLD signal in sgACC, mPFC, hippocampus, right amygdala, putamen, and somatomotor cortex regions. Ketamine-related changes in right insula BOLD signal were significantly associated with antidepressant response, further suggesting symptom-linked engagement of salience–reward integration circuitry. Across studies, ketamine doses ranged from 0.2 to 0.5 mg/kg, and imaging time points varied widely (during infusion to 2–48 hours, with a few extending to 7–10 days). These differences should be considered when interpreting cross-study patterns.^41^^,^^45^^–^^48^

### Findings from task-based fMRI

Task-based fMRI studies typically administered 0.5 mg/kg IV ketamine over 40 minutes, with scanning conducted either 2 hours post-infusion, 24 hours post-infusion, or 2 days post-infusion, depending on the paradigm.^49^^–^^52^ Importantly, not all task paradigms were reward-specific. Dot-probe and emotional face/emotion-processing tasks primarily index attentional bias and affective salience/emotion processing, which engage salience and affective control systems (eg insula, dorsal ACC, amygdala) and may influence reward processing indirectly. In contrast, reward-specific paradigms such as the monetary incentive delay (MID) task more directly probe reward anticipation and feedback processes typically involving striatal circuitry. Reward-related outcomes were assessed using tasks involving attentional bias to affective cues, explicit emotional processing, facial emotion perception, and monetary reward anticipation and receipt. Using the dot-probe test with emotional stimuli, ketamine produced significantly greater activation in the left middle occipital gyrus compared with placebo (pFWE <0.001), while reducing activation in the left temporal and inferior frontal cortices (pFWE <0.002).^50^ Across conditions, angry faces elicited greater activation than happy faces in bilateral dACC, putamen, thalamus, and posterior cingulate, but no main group effects were observed. In an emotional processing paradigm, ketamine compared with placebo led to widespread reductions in activity across bilateral frontal, temporal, precuneus, and posterior cingulate regions in patients with depression (pFWE <0.001).^51^ No regions showed increased activation post-ketamine in this group. In healthy controls, ketamine reduced activity in the bilateral cingulate cortex but increased activation in a large frontal cluster (pFWE <0.001). Reward anticipation and outcome processing were assessed using the MID task.^52^ Ketamine increased activity during the anticipation of win versus neutral trials across predefined reward-related ROIs (F1,36 = 9.261, p = .003), although effects in NAc and caudate during high-win anticipation did not survive correction for multiple comparisons. During the feedback phase of low-win trials, ketamine significantly increased activity in the NAc and putamen compared with placebo (pCORR = .008). Additional effects across ROIs were observed during no-win trials (F1,36 = 5.467, p < .001) but did not survive ROI-level correction. VTA activation during low-win feedback significantly correlated with plasma (2R,6R)-HNK levels 2 hours post-infusion (pFDR = .03). Emotional face processing tasks further revealed modulation of medial prefrontal and cingulate activity. During infusion, ketamine significantly reduced BOLD responses in the medial prefrontal cortex (mPFC) and posterior cingulate/precuneus compared with placebo (p < .05). Positive emotion processing further demonstrated ketamine-related modulation of striatal activity. In a task contrasting happy versus neutral faces, responses in the right caudate increased following ketamine, whereas neutral responses remained stable (cluster-level FWE p < .05).^49^ A significant interaction was also observed in the left middle frontal gyrus during negative emotion trials, though effects were less robust.

### Other imaging findings

In a phMRI study using an emotional faces paradigm, ketamine significantly increased BOLD signal in the subgenual ACC (sgACC) and rostral ACC (rACC) relative to placebo (all *p*FWE < 0.05).^53^ Responses followed a gradual increase that plateaued after approximately 24 minutes of infusion. Ketamine also increased activity in the mid and posterior cingulate cortex, bilateral thalamus, and caudate, peaking within the first 4 minutes of infusion. Using FDG-PET, Chen et al. (2017) reported that TRD patients receiving 0.5 mg/kg ketamine had significantly increased standardized uptake values (SUV) in the supplementary motor area and dorsal ACC compared with patients receiving 0.2 mg/kg (*p* = 0.014).^54^ No significant main effects of time or group were observed in ANOVA, though the increase in dACC SUV was statistically significant. In a PET study of SSRI-resistant depression using a 5-HT1B receptor ligand, ketamine was not associated with significant ROI-wide binding changes relative to placebo, but hippocampal binding increased after the first ketamine infusion (p = .036).^55^ Baseline ventral striatal 5-HT1B binding was negatively correlated with depressive symptom severity and also negatively correlated with magnitude of symptom reduction following ketamine, supporting a predictive association involving reward-related serotonergic markers.

In a diffusion MRI trial, ketamine showed no significant main effect on mean diffusivity (MD) change across examined regions at 24 hours, but regional MD changes moderated symptom outcomes.^56^ Decreases in MD in left BA10 and left amygdala were associated with greater improvement in depression scores in the ketamine group, indicating microstructural–clinical associations in prefrontal–limbic circuitry relevant to reward valuation and affect regulation. In contrast, increases in hippocampal MD were associated with greater improvement in MADRS within the ketamine group, suggesting region-specific directionality in microstructural associations^56^.

## Discussion

The present systematic review synthesizes evidence from functional neuroimaging studies to evaluate the associations between ketamine administration and neural reward processing in adults with MDD/TRD. In addressing our first objective, the included studies generally demonstrated associations between ketamine and alterations in neural activity or connectivity within ventral striatal, ACC, and prefrontal regions. Regarding our second objective, multimodal neuroimaging findings suggest that these neural patterns often co-occur with rapid antidepressant improvement; however, given the correlational nature of the data, these patterns should not be interpreted as direct evidence that ketamine causes specific circuit-level modifications or synaptic changes. Only a minority of studies directly measured anhedonia using validated scales such as SHAPS or TEPS, whereas most relied on global depression instruments. As a result, the reviewed findings should be understood as reflecting broader reward-related and/or antidepressant-associated processes, rather than definitive evidence of ketamine-specific anti-anhedonic mechanisms.

### Modulation of reward circuitry

Converging evidence indicates that subanesthetic dosing of ketamine is associated with increased neural activity in key reward-processing regions. Specifically, in a monetary incentive task, ketamine (0.5 mg/kg) was linked to significantly higher BOLD activation in the NAcc and putamen during reward feedback just 2 hours post-infusion, compared to placebo.^52^ Because symptom assessments and imaging time points varied across studies, these neural differences may occur prior to, alongside, or shortly before measurable clinical improvement, but they should not be interpreted as temporally proving symptom change. Task-based fMRI studies consistently indicate that ketamine can acutely normalize or heighten responses in ventral striatal regions that are often blunted in depression, such as enhancing neural reactivity to positive/reward cues, which may relate to, but do not definitively establish, ketamine’s anti-anhedonic properties.^52^^,^^62^ Ketamine was also associated with differences in FC within the broader reward network, particularly between the PFC and striatum, similar to findings in other study designs.^48^^,^^63^ Resting-state fMRI studies 24 hours after ketamine show increased connectivity in fronto-striatal circuits, with responders exhibiting increased connectivity between the lateral PFC and striatal regions like the caudate.^63^ In persons with depression, ketamine increased fronto-striatal connectivity (interpreted as a normalization of circuit hypoactivity associated with anhedonia), whereas healthy volunteers showed slight connectivity reductions post-ketamine, suggesting an association between ketamine treatment and modulation of cortico-striatal loops implicated in motivation and reward processing.

In addition, PET imaging provides complementary evidence wherein ketamine was associated with region-specific changes in 5-HT1B receptor binding (eg hippocampus), and baseline ventral striatal 5-HT1B binding was associated with symptom severity and symptom change in depressed patients.^55^ The aforementioned electrophysiological findings accord with the fMRI results by highlighting ketamine’s rapid enhancement of neural network responsiveness. Taken together, task-activation studies, connectivity analyses, and molecular imaging indicate that ketamine is associated with modulation of reward circuitry, which supports emerging literature suggesting that modulation of the positive valence system may be a mechanistic target for personalized treatment strategies in depression.^34^

It is important to note that the observed variability across studies may be partly attributable to heterogeneity in ketamine dosing (0.2–0.5 mg/kg), and timing of imaging. Differences in study populations—particularly TRD versus non-resistant MDD and the inclusion of healthy controls—likely further contribute to divergent findings. Analytic choices, including ROI-based versus whole-brain approaches, also shape the reported neural patterns.

### Biological mechanisms of ketamine’s action

Findings indicate that ketamine is associated with increased availability of serotonin 1B receptors in depressed patients.^55^ This is mechanistically interesting because 5-HT₁_B receptors are autoreceptors and heteroreceptors that modulate neurotransmitter release: their activation tends to inhibit serotonin release but facilitates dopamine release in the striatum.^64^ Increased FC between the mPFC and the NAcc and heightened ventral striatal activation during reward tasks have been interpreted as potentially reflecting underlying synaptic potentiation driven by glutamatergic plasticity.^42^^,^^47^^,^^48^^,^^50^^,^^52^ Electrophysiological evidence further supports this interpretation, as ketamine-induced increases in gamma-band oscillations—markers of cortical excitation and local circuit reorganization—were observed shortly after infusion.^43^^,^^46^^,^^65^

On a systems level, ketamine may act as a “network reset” agent, disrupting pathological brain states commonly observed in depression. The acute network desynchronization observed in EEG and fMRI studies immediately following ketamine infusion disrupts maladaptive connectivity patterns, such as hyperconnectivity of the default mode network and hypoconnectivity of reward circuits.^46^^,^^65^ Over time, as NMDA receptor blockade subsides, brain networks appear to reconfigure into healthier, more interconnected states characterized by restored fronto-striatal and executive network integration.^66^ Additionally, research has found that higher plasma levels of the ketamine metabolite HNK were associated with greater activation of the VTA, consistent with HNK’s role as an AMPA receptor facilitator promoting continued activation of dopaminergic reward pathways.^52^^,^^67^ Ketamine’s ability to rapidly reactivate dormant synapses, enhance neurotrophic signaling, amplify dopaminergic activity, and reconfigure network dynamics offers a multi-dimensional mechanism that distinguishes it from traditional monoaminergic antidepressants and explains its unique efficacy in rapidly restoring interest, motivation, and pleasure in depression. While preclinical models robustly demonstrate mechanisms such as AMPA-mediated synaptic strengthening, BDNF-dependent neuroplasticity, and dendritic spine formation, these cellular processes cannot be directly measured using human neuroimaging techniques. Thus, references to “synaptic potentiation” or “network reconfiguration” in humans should be understood as theoretical interpretations, supported by animal literature and consistent with observed human imaging correlates, but not directly demonstrated in the included studies.

### Limitations

A crucial consideration is the presence of inconsistent methods across imaging modalities. Each modality and task address different aspects of neural function, which complicates direct comparison. For example, a monetary reward task may specifically index ventral striatal phasic activity, whereas resting connectivity focuses on intrinsic synchronization.^67^ Divergence in results may arise due to one capturing evoked activity and the other spontaneous activity. In addition, analytic choices vary, wherein some analyses were ROI-driven (regions like NAcc or sgACC), while others were whole-brain or connectome-based. Other methodological differences that may affect our interpretation of the results include the sociodemographic differences (eg illness duration, comorbidities, additional antidepressant treatments) in the enrolled population. Most imaging assessments were acute; limited data exist on whether these brain changes correlate with longer-term outcomes (eg 1–2 weeks or relapse timing). The short follow-ups (often 24 h or 7 days at most) are a limitation if we aim to tie neural changes to lasting clinical remission. A formal assessment of publication bias could not be conducted due to the limited number of included studies and substantial heterogeneity in imaging modalities and task paradigms, which violate the assumptions of statistical bias tests such as funnel plots or Egger’s regression. As a result, the presence of publication bias cannot be ruled out. Neuroimaging studies in particular are susceptible to small-sample reporting and selective publication of significant findings, which should be considered when interpreting the results. Finally, the protocol was not prospectively registered. Although our eligibility criteria and outcomes were established a priori and did not change during the review, the lack of public registration may reduce transparency relative to best-practice standards.

### Future directions

Future research should involve larger, adequately controlled neuroimaging trials and utilize machine learning models to predict ketamine response for personalized treatments.^68^ Longitudinal studies tracking multiple time points are needed to elucidate ketamine’s sustained neural effects. Combining PET, fMRI, EEG, and MR spectroscopy can clarify interactions across molecular, functional, and electrophysiological domains.^38^^,^^46^ Future studies should prioritize anhedonia and reward function as primary outcomes, integrating behavioral measures of real-world reward experiences to better translate brain changes into clinical recovery.^62^

## Conclusion

This systematic review found that ketamine administration is associated with neurofunctional changes in reward-related circuitry in adults with MDD/TRD, most consistently involving the ventral striatum/NAc and connected ACC–prefrontal networks. Given heterogeneity in methods and timing—and because few studies directly measured anhedonia—these findings should be interpreted as reflecting broader reward- and antidepressant-associated processes rather than definitive ketamine-specific anti-anhedonic mechanisms. Larger longitudinal neuroimaging studies that prioritize validated anhedonia outcomes are needed to clarify the consistency, time course, and clinical relevance of these associations.^25^

## Supporting information

10.1017/S109285292610087X.sm001 Faisal et al. Supplementary Material Faisal et al. Supplementary Material

## Supplementary material

The supplementary material for this article can be found at http://doi.org/10.1017/S109285292610087X.
