# Supplementary material for: Effect of ketamine on reward processing in depressive disorders: a systematic review of neuroimaging studies
Source: CNS Spectr. 2026 Mar 10;31(1):e9. doi: 10.1017/S109285292610087X (PMC13287819; doi:10.1017/S109285292610087X)
Supplement: Faisal et al. Supplementary Material [file S109285292610087Xsup001.docx]

Supplementary Appendix 1. Full Search Strategies for All Databases

**Ovid Embase**

| # | Searches | Results | Type |
| --- | --- | --- | --- |
| 1 | exp depression/ or exp depressive disorder/ or exp depressive disorder, major/ or exp depressive disorder, treatment-resistant/ | 726785 | Advanced |
| 2 | (depress* or "major depressive disorder" or MDD or "clinical depression" or "unipolar depression" or "refractory depression" or anhedonia or "loss of pleasure" or "reduced reward sensitivity" or "treatment-resistant depression" or TRD or "persistent depressive disorder" or dysthymia or "chronic depression" or "affective disorders" or "mood disorders" or melancholia).mp. | 1139764 | Advanced |
| 3 | 1 or 2 | 1186644 | Advanced |
| 4 | exp ketamine/ or exp esketamine/ or exp S-ketamine/ or exp NMDA antagonists/ | 72531 | Advanced |
| 5 | (ketamine or "s-ketamine" or esketamine or "r-ketamine" or "NMDA antagonist" or "N-methyl-D-aspartate antagonist" or "intravenous ketamine" or "IV ketamine" or "ketamine infusion" or "ketamine therapy" or "ketamine treatment" or "intranasal esketamine" or Spravato or "glutamate modulator").mp. | 80196 | Advanced |
| 6 | 4 or 5 | 80196 | Advanced |
| 7 | exp reward/ or exp reinforcement, psychology/ or exp reward circuitry/ | 149177 | Advanced |
| 8 | (reward process* or "reward circuit*" or "reward system" or "dopaminergic system" or dopamine or "dopamine signaling" or "nucleus accumbens" or "ventral striatum" or "mesolimbic pathway*" or "reward response" or "reinforcement learning" or motivation or "pleasure response" or "hedonic tone" or "reward sensitivity" or "reward-related behavior" or "reward anticipation" or "neural reward mechanisms").mp. | 499559 | Advanced |
| 9 | 7 or 8 | 610511 | Advanced |
| 10 | exp neuroimaging/ or exp functional MRI/ or exp positron emission tomography/ | 531002 | Advanced |
| 11 | (fMRI or PET or "functional MRI" or "positron emission tomography" or neuroimaging or "brain imaging" or EEG or MEG or "functional connectivity" or "magnetic resonance spectroscopy" or "resting-state connectivity" or "neurofunctional imaging" or "brain mapping" or "structural MRI" or DTI or "diffusion tensor imaging").mp. | 1061118 | Advanced |
| 12 | 10 or 11 | 1072890 | Advanced |
| 13 | 3 and 6 and 9 and 12 | 237 | Advanced |
| 14 | limit 13 to (human and yr="2000 -Current") | 193 | Advanced |

**Ovid Medline**

| # | Searches | Results | Type |
| --- | --- | --- | --- |
| 1 | exp depression/ or exp depressive disorder/ or exp depressive disorder, major/ or exp depressive disorder, treatment-resistant/ | 276767 | Advanced |
| 2 | (depress* or "major depressive disorder" or MDD or "clinical depression" or "unipolar depression" or "refractory depression" or anhedonia or "loss of pleasure" or "reduced reward sensitivity" or "treatment-resistant depression" or TRD or "persistent depressive disorder" or dysthymia or "chronic depression" or "affective disorders" or "mood disorders" or melancholia).mp. | 732354 | Advanced |
| 3 | 1 or 2 | 733030 | Advanced |
| 4 | exp ketamine/ or exp esketamine/ or exp S-ketamine/ or exp NMDA antagonists/ | 16175 | Advanced |
| 5 | (ketamine or "s-ketamine" or esketamine or "r-ketamine" or "NMDA antagonist" or "N-methyl-D-aspartate antagonist" or "intravenous ketamine" or "IV ketamine" or "ketamine infusion" or "ketamine therapy" or "ketamine treatment" or "intranasal esketamine" or Spravato or "glutamate modulator").mp. | 29549 | Advanced |
| 6 | 4 or 5 | 29549 | Advanced |
| 7 | exp reward/ or exp reinforcement, psychology/ or exp reward circuitry/ | 64549 | Advanced |
| 8 | (reward process* or "reward circuit*" or "reward system" or "dopaminergic system" or dopamine or "dopamine signaling" or "nucleus accumbens" or "ventral striatum" or "mesolimbic pathway*" or "reward response" or "reinforcement learning" or motivation or "pleasure response" or "hedonic tone" or "reward sensitivity" or "reward-related behavior" or "reward anticipation" or "neural reward mechanisms").mp. | 357944 | Advanced |
| 9 | 7 or 8 | 401499 | Advanced |
| 10 | exp neuroimaging/ or exp functional MRI/ or exp positron emission tomography/ | 752354 | Advanced |
| 11 | (fMRI or PET or "functional MRI" or "positron emission tomography" or neuroimaging or "brain imaging" or EEG or MEG or "functional connectivity" or "magnetic resonance spectroscopy" or "resting-state connectivity" or "neurofunctional imaging" or "brain mapping" or "structural MRI" or DTI or "diffusion tensor imaging").mp. | 693885 | Advanced |
| 12 | 10 or 11 | 1160585 | Advanced |
| 13 | 3 and 6 and 9 and 12 | 43 | Advanced |
| 14 | limit 13 to (humans and yr="2000 -Current") | 27 | Advanced |

**Ovid PsycINFO**

| # | Searches | Results | Type |
| --- | --- | --- | --- |
| 1 | exp depressive disorder/ or exp major depression/ or exp dysthymic disorder/ or exp anhedonia/ | 193661 | Advanced |
| 2 | (depress* or "major depressive disorder" or MDD or "clinical depression" or "unipolar depression" or "refractory depression" or anhedonia or "loss of pleasure" or "reduced reward sensitivity" or "treatment-resistant depression" or TRD or "persistent depressive disorder" or dysthymia or "chronic depression" or "affective disorders" or "mood disorders" or "melancholia").mp. | 491378 | Advanced |
| 3 | 1 or 2 | 492029 | Advanced |
| 4 | exp ketamine/ or exp esketamine/ or exp NMDA receptor antagonists/ | 3429 | Advanced |
| 5 | (ketamine or "s-ketamine" or esketamine or "r-ketamine" or "NMDA antagonist" or "N-methyl-D-aspartate antagonist" or "intravenous ketamine" or "IV ketamine" or "ketamine infusion" or "ketamine therapy" or "ketamine treatment" or "intranasal esketamine" or Spravato or "glutamate modulator").mp. | 6467 | Advanced |
| 6 | 4 or 5 | 6467 | Advanced |
| 7 | exp reward/ or exp reinforcement, psychology/ or exp motivation/ | 205020 | Advanced |
| 8 | (reward process* or "reward circuit*" or "reward system" or "dopaminergic system" or dopamine or "dopamine signaling" or "nucleus accumbens" or "ventral striatum" or "mesolimbic pathway*" or "reward response" or "reinforcement learning" or motivation or "pleasure response" or "hedonic tone" or "reward sensitivity" or "reward-related behavior" or "reward anticipation" or "neural reward mechanisms").mp. | 245391 | Advanced |
| 9 | 7 or 8 | 351194 | Advanced |
| 10 | exp neuroimaging/ or exp functional neuroimaging/ or exp functional MRI/ or exp positron emission tomography/ | 127307 | Advanced |
| 11 | (fMRI or PET or "functional MRI" or "positron emission tomography" or neuroimaging or "brain imaging" or EEG or MEG or "functional connectivity" or "magnetic resonance spectroscopy" or "resting-state connectivity" or "neurofunctional imaging" or "brain mapping" or "structural MRI" or DTI or "diffusion tensor imaging").mp. | 186498 | Advanced |
| 12 | 10 or 11 | 211351 | Advanced |
| 13 | 3 and 6 and 9 and 12 | 26 | Advanced |
| 14 | Limit 13 to (human and yr=”2000 -Current”) | 19 | Advanced |

**Wiley Cochrane Library**

| # | Searches | Results | Type |
| --- | --- | --- | --- |
| 1 | (depress* OR "major depressive disorder" OR MDD OR "clinical depression" OR "unipolar depression" OR "refractory depression" OR anhedonia OR "loss of pleasure" OR "reduced reward sensitivity" OR "treatment-resistant depression" OR TRD OR "persistent depressive disorder" OR dysthymia OR "chronic depression" OR "affective disorders" OR "mood disorders" OR melancholia):ti,ab,kw | 125256 | Advanced |
| 2 | ketamine OR "s-ketamine" OR esketamine OR "r-ketamine" OR "NMDA antagonist" OR "N-methyl-D-aspartate antagonist" OR "intravenous ketamine" OR "IV ketamine" OR "ketamine infusion" OR "ketamine therapy" OR "ketamine treatment" OR "intranasal esketamine" OR Spravato OR "glutamate modulator" | 9246 | Advanced |
| 3 | "reward process" OR "reward circuit" OR "reward system" OR "dopaminergic system" OR dopamine OR "dopamine signaling" OR "nucleus accumbens" OR "ventral striatum" OR "mesolimbic pathway" OR "reward response" OR "reinforcement learning" OR motivation OR "pleasure response" OR "hedonic tone" OR "reward sensitivity" OR "reward-related behavior" OR "reward anticipation" OR "neural reward mechanisms" | 30231 | Advanced |
| 4 | fMRI OR PET OR "functional MRI" OR "positron emission tomography" OR neuroimaging OR "brain imaging" OR EEG OR MEG OR "functional connectivity" OR "magnetic resonance spectroscopy" OR "resting-state connectivity" OR "neurofunctional imaging" OR "brain mapping" OR "structural MRI" OR DTI OR "diffusion tensor imaging" | 39480 | Advanced |
| 5 | #1 AND #2 AND #3 AND #4 | 15 | Advanced |

**Scopus**

TITLE-ABS-KEY ( depress* OR "major depressive disorder" OR mdd OR "clinical depression" OR "unipolar depression" OR "refractory depression" OR anhedonia OR "loss of pleasure" OR "reduced reward sensitivity" OR "treatment-resistant depression" OR trd OR "persistent depressive disorder" OR dysthymia OR "chronic depression" OR "affective disorders" OR "mood disorders" OR melancholia ) AND TITLE-ABS-KEY ( ketamine OR "s-ketamine" OR esketamine OR "r-ketamine" OR "NMDA antagonist" OR "N-methyl-D-aspartate antagonist" OR "intravenous ketamine" OR "IV ketamine" OR "ketamine infusion" OR "ketamine therapy" OR "ketamine treatment" OR "intranasal esketamine" OR spravato OR "glutamate modulator" ) AND TITLE-ABS-KEY ( "reward process" OR "reward circuit" OR "reward system" OR "dopaminergic system" OR dopamine OR "dopamine signaling" OR "nucleus accumbens" OR "ventral striatum" OR "mesolimbic pathway" OR "reward response" OR "reinforcement learning" OR motivation OR "pleasure response" OR "hedonic tone" OR "reward sensitivity" OR "reward-related behavior" OR "reward anticipation" OR "neural reward mechanisms" ) AND TITLE-ABS-KEY ( fmri OR pet OR "functional MRI" OR "positron emission tomography" OR neuroimaging OR "brain imaging" OR eeg OR meg OR "functional connectivity" OR "magnetic resonance spectroscopy" OR "resting-state connectivity" OR "neurofunctional imaging" OR "brain mapping" OR "structural MRI" OR dti OR "diffusion tensor imaging" ) AND PUBYEAR > 2002 AND PUBYEAR < 2026 AND ( LIMIT-TO ( EXACTKEYWORD , "Humans" ) )

**Web of Science**

TS=(depress* OR "major depressive disorder" OR MDD OR "clinical depression" OR "unipolar depression" OR "refractory depression" OR anhedonia OR "loss of pleasure" OR "reduced reward sensitivity" OR "treatment-resistant depression" OR TRD OR "persistent depressive disorder" OR dysthymia OR "chronic depression" OR "affective disorders" OR "mood disorders" OR melancholia) AND TS=(ketamine OR "s-ketamine" OR esketamine OR "r-ketamine" OR "NMDA antagonist" OR "N-methyl-D-aspartate antagonist" OR "intravenous ketamine" OR "IV ketamine" OR "ketamine infusion" OR "ketamine therapy" OR "ketamine treatment" OR "intranasal esketamine" OR spianato OR "glutamate modulator") AND TS=("reward process" OR "reward circuit" OR "reward system" OR "dopaminergic system" OR dopamine OR "dopamine signaling" OR "nucleus accumbens" OR "ventral striatum" OR "mesolimbic pathway" OR "reward response" OR "reinforcement learning" OR motivation OR "pleasure response" OR "hedonic tone" OR "reward sensitivity" OR "reward-related behavior" OR "reward anticipation" OR "neural reward mechanisms") AND TS=(fMRI OR PET OR "functional MRI" OR "positron emission tomography" OR neuroimaging OR "brain imaging" OR EEG OR MEG OR "functional connectivity" OR "magnetic resonance spectroscopy" OR "resting-state connectivity" OR "neurofunctional imaging" OR "brain mapping" OR "structural MRI" OR DTI OR "diffusion tensor imaging")

**Google Scholar**

("Major Depressive Disorder" OR "MDD" OR "clinical depression" OR "treatment-resistant depression" OR "TRD")

AND ("Ketamine" OR "esketamine" OR "r-ketamine" OR "intranasal esketamine" OR "Spravato" OR "NMDA antagonist")

AND ("reward system" OR "dopaminergic system" OR "nucleus accumbens" OR "ventral striatum" OR "reinforcement learning")

AND ("fMRI" OR "PET scan" OR "neuroimaging" OR "functional MRI" OR "brain imaging") 🡪 3,550 results

Supplementary Table 1. PRISMA 2020 Checklist

| **Section and Topic** | **Item #** | **Checklist item** | **Location where item is reported** |
| --- | --- | --- | --- |
| **TITLE** | | |  |
| Title | 1 | Identify the report as a systematic review. | Page 1 – Title Page |
| **ABSTRACT** | | |  |
| Abstract | 2 | See the PRISMA 2020 for Abstracts checklist. | Page 2 – Structured Abstract |
| **INTRODUCTION** | | |  |
| Rationale | 3 | Describe the rationale for the review in the context of existing knowledge. | Page 3,4 – Introduction, paragraph 1–2 |
| Objectives | 4 | Provide an explicit statement of the objective(s) or question(s) the review addresses. | Page 5 – End of Introduction |
| **METHODS** | | |  |
| Eligibility criteria | 5 | Specify the inclusion and exclusion criteria for the review and how studies were grouped for the syntheses. | Methods – Page 5 |
| Information sources | 6 | Specify all databases, registers, websites, organisations, reference lists and other sources searched or consulted to identify studies. Specify the date when each source was last searched or consulted. | Methods – Page 5 + Supplementary Appendix 1 |
| Search strategy | 7 | Present the full search strategies for all databases, registers and websites, including any filters and limits used. | Supplementary Appendix 1 |
| Selection process | 8 | Specify the methods used to decide whether a study met the inclusion criteria of the review, including how many reviewers screened each record and each report retrieved, whether they worked independently, and if applicable, details of automation tools used in the process. | Methods – Page 6 |
| Data collection process | 9 | Specify the methods used to collect data from reports, including how many reviewers collected data from each report, whether they worked independently, any processes for obtaining or confirming data from study investigators, and if applicable, details of automation tools used in the process. | Methods – Page 6 |
| Data items | 10a | List and define all outcomes for which data were sought. Specify whether all results that were compatible with each outcome domain in each study were sought (e.g. for all measures, time points, analyses), and if not, the methods used to decide which results to collect. | Methods – Page 6,7 & Title Page |
|  | 10b | List and define all other variables for which data were sought (e.g. participant and intervention characteristics, funding sources). Describe any assumptions made about any missing or unclear information. |  |
| Study risk of bias assessment | 11 | Specify the methods used to assess risk of bias in the included studies, including details of the tool(s) used, how many reviewers assessed each study and whether they worked independently, and if applicable, details of automation tools used in the process. | Results – Page 6 |
| Effect measures | 12 | Specify for each outcome the effect measure(s) (e.g. risk ratio, mean difference) used in the synthesis or presentation of results. | Results – Page 7,8 |
| Synthesis methods | 13a | Describe the processes used to decide which studies were eligible for each synthesis (e.g. tabulating the study intervention characteristics and comparing against the planned groups for each synthesis (item #5)). | Methods – Page 6 |
|  | 13b | Describe any methods required to prepare the data for presentation or synthesis, such as handling of missing summary statistics, or data conversions. | Methods – Page 6 |
|  | 13c | Describe any methods used to tabulate or visually display results of individual studies and syntheses. | Methods – Page 6 |
|  | 13d | Describe any methods used to synthesize results and provide a rationale for the choice(s). If meta-analysis was performed, describe the model(s), method(s) to identify the presence and extent of statistical heterogeneity, and software package(s) used. | Methods – Page 6 |
|  | 13e | Describe any methods used to explore possible causes of heterogeneity among study results (e.g. subgroup analysis, meta-regression). | N/A |
|  | 13f | Describe any sensitivity analyses conducted to assess robustness of the synthesized results. | N/A |
| Reporting bias assessment | 14 | Describe any methods used to assess risk of bias due to missing results in a synthesis (arising from reporting biases). | Results – Page 6 |
| Certainty assessment | 15 | Describe any methods used to assess certainty (or confidence) in the body of evidence for an outcome. | N/A |
| **RESULTS** | | |  |
| Study selection | 16a | Describe the results of the search and selection process, from the number of records identified in the search to the number of studies included in the review, ideally using a flow diagram. | Results – Page 7; Figure 1 (PRISMA Flowchart) |
|  | 16b | Cite studies that might appear to meet the inclusion criteria, but which were excluded, and explain why they were excluded. | Results – Page 7 |
| Study characteristics | 17 | Cite each included study and present its characteristics. | Reference List – Pages 12-19 |
| Risk of bias in studies | 18 | Present assessments of risk of bias for each included study. | Figure 2 |
| Results of individual studies | 19 | For all outcomes, present, for each study: (a) summary statistics for each group (where appropriate) and (b) an effect estimate and its precision (e.g. confidence/credible interval), ideally using structured tables or plots. | N/A |
| Results of syntheses | 20a | For each synthesis, briefly summarise the characteristics and risk of bias among contributing studies. | Results – Page 6 |
|  | 20b | Present results of all statistical syntheses conducted. If meta-analysis was done, present for each the summary estimate and its precision (e.g. confidence/credible interval) and measures of statistical heterogeneity. If comparing groups, describe the direction of the effect. | N/A |
|  | 20c | Present results of all investigations of possible causes of heterogeneity among study results. | N/A |
|  | 20d | Present results of all sensitivity analyses conducted to assess the robustness of the synthesized results. | N/A |
| Reporting biases | 21 | Present assessments of risk of bias due to missing results (arising from reporting biases) for each synthesis assessed. | Results – Page 6 |
| Certainty of evidence | 22 | Present assessments of certainty (or confidence) in the body of evidence for each outcome assessed. | N/A |
| **DISCUSSION** | | |  |
| Discussion | 23a | Provide a general interpretation of the results in the context of other evidence. | Discussion – Pages 9-11 |
|  | 23b | Discuss any limitations of the evidence included in the review. | Discussion – Pages 10,11 |
|  | 23c | Discuss any limitations of the review processes used. | Discussion – Pages 10,11 |
|  | 23d | Discuss implications of the results for practice, policy, and future research. | Discussion – Pages 11 |
| **OTHER INFORMATION** | | |  |
| Registration and protocol | 24a | Provide registration information for the review, including register name and registration number, or state that the review was not registered. | N/A |
|  | 24b | Indicate where the review protocol can be accessed, or state that a protocol was not prepared. | N/A |
|  | 24c | Describe and explain any amendments to information provided at registration or in the protocol. | N/A |
| Support | 25 | Describe sources of financial or non-financial support for the review, and the role of the funders or sponsors in the review. | Title page |
| Competing interests | 26 | Declare any competing interests of review authors. |  |
| Availability of data, code and other materials | 27 | Report which of the following are publicly available and where they can be found: template data collection forms; data extracted from included studies; data used for all analyses; analytic code; any other materials used in the review. | Reference List |

*From:*  Page MJ, McKenzie JE, Bossuyt PM, Boutron I, Hoffmann TC, Mulrow CD, et al. The PRISMA 2020 statement: an updated guideline for reporting systematic reviews. BMJ 2021;372:n71. doi: 10.1136/bmj.n71. This work is licensed under CC BY 4.0. To view a copy of this license, visit <https://creativecommons.org/licenses/by/4.0/>
